# Adolescent circadian sleep health: a review of multimodal assessment strategies and clinical implications

**DOI:** 10.3389/frsle.2026.1826938

**Published:** 2026-08-19

**Authors:** Jamon M. Jex, Alyssa Larson Downs, Sarah Kamhout, Isabella D. Wright, Andrew Wright, Victoria Zhang, Kara McRae Duraccio

**Affiliations:** Department of Psychology, Brigham Young University, Provo, UT, United States

**Keywords:** adolescents, chronotype, circadian alignment, circadian misalignment, circadian sleep health, circadian timing, multimodal assessment, sleep health

## Abstract

The adolescent developmental period is marked by increased social and academic demands, as well as biological changes that lead to profound shifts in sleep and circadian timing. Circadian misalignment, short sleep duration, and irregular sleep patterns are prevalent among adolescents and are linked to a wide range of negative outcomes, including impaired emotional regulation, diminished academic performance, and increased cardiometabolic disease risk. While efforts to promote adolescent sleep health have grown, the multidimensional nature of circadian sleep health remains underassessed in both research and clinical contexts. This review provides conceptual definitions of key circadian sleep health concepts, synthesizing current best practices for measuring circadian sleep health in adolescents across self-report, behavioral, and physiological domains. We organize assessment strategies around three key circadian constructs—chronotype, circadian timing, and circadian alignment—and describe validated tools for each. In addition, we explore measurements of contextual factors influencing circadian function, such as light exposure, meal and exercise timing, pubertal status, and genetic predispositions. Throughout this review, we emphasize the value of a multimodal approach that combines subjective experience with objective biological and behavioral data to improve clinical utility and advance the understanding of circadian sleep health. We conclude by outlining directions for future research, including the need for validated, developmentally appropriate tools and the integration of wearable technologies to improve accessibility and clinical utility. By consolidating core concepts and methodological tools, this review aims to assist researchers and clinicians selecting appropriate assessment strategies and tailoring interventions to improve adolescent circadian sleep health.

## 1 Introduction

Sleep is a crucial aspect of adolescent wellbeing, affecting various neurological, cognitive, emotional, metabolic, and immunological functions (Meyer et al., 2022). Inadequate sleep duration during adolescence has been associated with various negative health risks, including worsened emotional functioning, decreased academic performance, and increased risk-taking (Duraccio et al., 2023; Moore et al., 2009; Roberts et al., 2001). Furthermore, short sleep duration, poor sleep timing, and low sleep quality are also associated with increased risk for cardiometabolic disease and obesity via the increased sedentary behavior, poor food choices, and higher caloric intake with which they are associated (Duraccio et al., 2019; Morales-Ghinaglia and Fernandez-Mendoza, 2023; Okoli et al., 2021). Alternatively, adolescents who receive adequate, well-timed sleep appear to have enhanced attention, learning, working memory, memory consolidation, creativity, executive function, emotional processing, and positive mood compared to those who do not (Brand and Kirov, 2011; Lo et al., 2016). Due to the vast neurocognitive and psychosocial impacts of sleep health, sleep promotion within adolescent populations is crucial for healthy development.

Sleep promotion is particularly important during the adolescent phase of development due to various biopsychosocial factors, outlined in the Perfect Storm model (Carskadon, 2011; Crowley et al., 2018), which place adolescents at greater risk of poor circadian sleep health. These factors include biological shifts in circadian timing toward later sleep onset and a slower accumulation of homeostatic sleep pressure, paired with socio-cultural factors such as early school start times and late-evening social engagements, homework, or technology use (Carskadon, 2011; Crowley et al., 2018). The combination of delayed biological sleep pressure, greater bedtime autonomy, and early school start times contributes to both short and ill-timed sleep in adolescents (Carskadon, 2011). National data suggest that U.S. adolescents average approximately 7 h of sleep per night (Giddens et al., 2022; Maslowsky and Ozer, 2014), below the American Academy of Pediatrics' recommendation of 8–10 h (Paruthi et al., 2016). This chronic weekday sleep deprivation is often compensated by later and longer periods of sleep on weekends, a phenomenon known as social jetlag (Carskadon, 2011; Crowley et al., 2014). Although adolescents often sleep longer on weekends to compensate for weekday sleep loss, social-jetlagged patterns of weekend catch-up sleep do not consistently improve perceived sleep quality and may sometimes be associated with worse subjective wellbeing (Tonetti et al., 2022). Greater variability in sleep timing between weekdays and weekends also disrupts circadian and metabolic rhythms and is associated with negative effects on physical and mental health (Dolsen et al., 2018; He et al., 2015; Huang et al., 2024; Kwon et al., 2025; Magnusdottir et al., 2024; Mathew et al., 2023; Morales-Ghinaglia and Fernandez-Mendoza, 2023). Therefore, adolescent health is impacted not only by the *amount* of sleep obtained, but also by the *timing* of when sleep occurs.

Distinguishing between sleep duration (how long one sleeps) and sleep timing (when one sleeps) highlights the multidimensional nature of sleep health (Buysse, 2014). Sleep health has been broadly conceptualized as a pattern of sleep and wakefulness that aligns with individual, social, and environmental demands while supporting both physical and mental wellbeing (Buysse, 2014). Beyond simple measures and interventions targeting sleep duration, this multidimensional perspective highlights the value of examining multiple domains of sleep in both research and clinical contexts. These various dimensions of sleep health are conceptually distinct, despite often co-occurring. For example, an adolescent may report feeling tired after a demanding school day and experience fatigue reflected by reduced physical or cognitive performance following chronic sleep restriction yet not exhibit elevated physiological sleep pressure or objective sleepiness on laboratory assessment. Recognizing these distinctions is important because each construct may arise from different underlying mechanisms and require different approaches to assessment and intervention. To provide a clearer foundation for distinguishing among these overlapping (but conceptually distinct) constructs, Table 1 summarizes common definitions of key sleep health dimensions and related experiences.

**Table 1:** Sleep health dimensions and definitions.

Dimension	Definition
Sleep duration	The amount of time between sleep onset and waking; actual time asleep (Dewald et al., 2010).
Sleep quality	Subjective indices of how sleep is experienced (i.e., the degree to which an individual feels rested and satisfied upon waking; Dewald et al., 2010).
Sleep timing	Time of day during which sleep occurs, measured with bedtime, wake-up time, or midpoint of sleep (Dutil et al., 2022).
Sleep regularity	Amount of daily consistency in sleep-wake timing (Windred et al., 2024); day-to-day changes in sleep midpoint (Morales-Ghinaglia and Fernandez-Mendoza, 2023).
Sleep variability	Day-to-day changes in sleep duration (Morales-Ghinaglia and Fernandez-Mendoza, 2023).
Sleep efficiency	The proportion of total sleep duration to time spent in bed attempting to sleep (excluding non-sleep activities done in bed, such as reading, texting, talking, etc.; Reed and Sacco, 2016).
Sleep onset latency	The amount of time required to transition from full wakefulness upon attempting to sleep to sleep onset; how long it takes an individual to “fall asleep” (Nixon et al., 2009).
Sleep offset latency	The amount of time between final awakening and getting out of bed (Chang et al., 2009).
Wake after sleep onset (WASO)	The amount of time an individual is awake between sleep onset and the end of their sleep period (sleep offset; Chase et al., 2022).
Sleep satisfaction	The level of an individual's perceived satisfaction with their sleep; often tied to sleep quantity and quality (Ohayon et al., 2018).
Sleepiness/sleep tendency	An involuntary physiological symptom of sleep pressure, distinct from subjective desire for rest; a reflection of state-dependent sleep drives in the body on cognitive and psychomotor performance, mood, motivation, and other systems (Carskadon and Dement, 1982; Shen et al., 2006).
Fatigue	A multidimensional construct that reflects a decline in the ability to sustain attention, effort, performance or alertness due to a depletion of energy and necessary neurochemical substrates (Aaronson et al., 1999; Skau et al., 2021).
Tiredness	A subjective feeling of need for rest (Skau et al., 2021).
Alertness	Physiological symptom of the lack of sleep pressure; a reflection of wake drives in the body on cognitive and psychomotor performance, mood, motivation, and other systems (Shen et al., 2006).
Daytime functioning	Negative or positive consequences of sleep behavior on cognitive, emotional, social, or energy function; negative consequences due to lack of sleep include fatigue, excessive sleepiness, irritability, mood swings (Ohayon et al., 2012).

This table highlights core dimensions but may not encapsulate all possible sleep dimensions that evolve as sleep science progresses.

**Figure 1:**
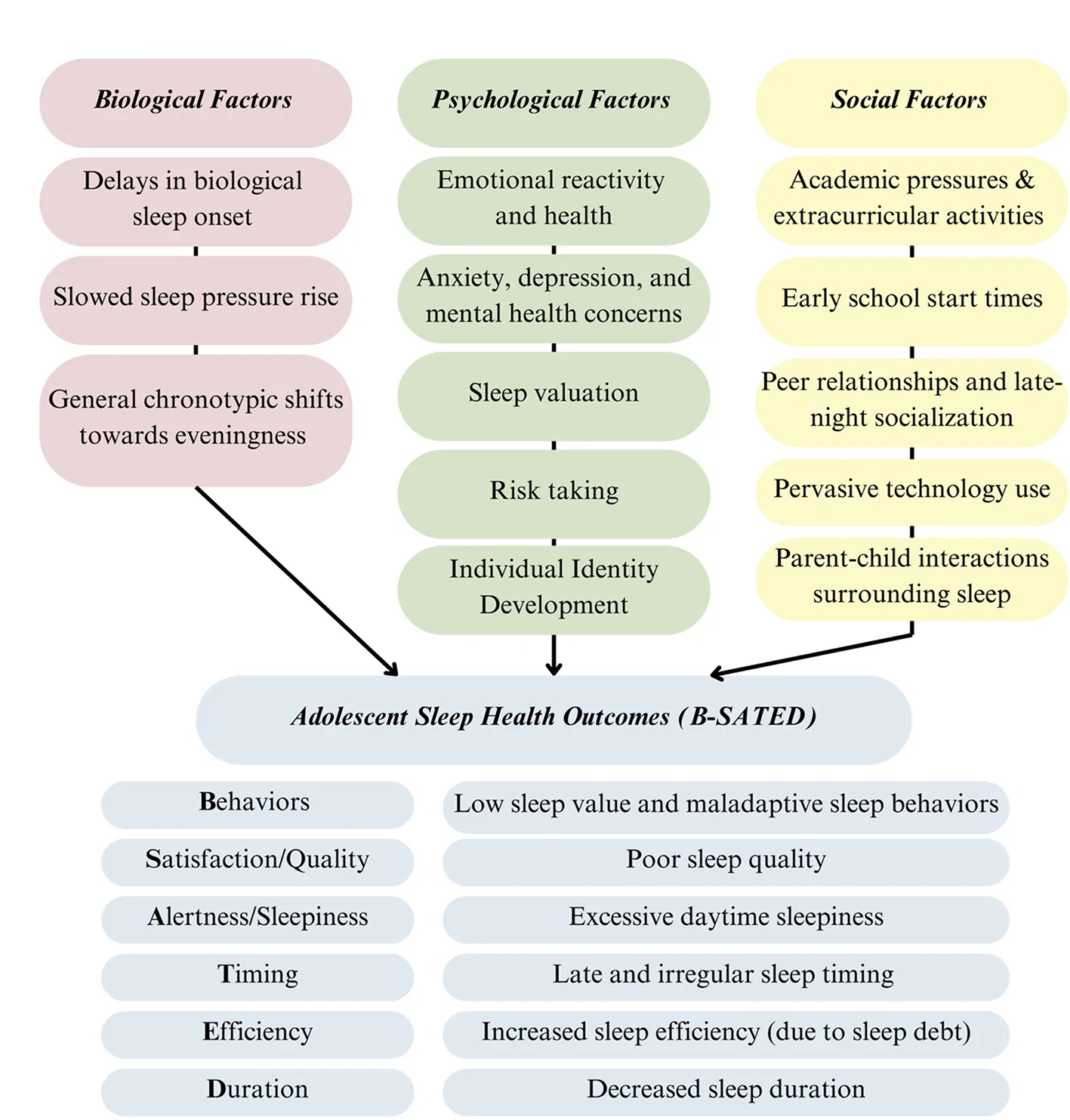
Biopsychosocial factors influencing adolescent sleep health outcomes. Adapted from Meltzer et al. (2021).

Despite the wide variety of sleep health dimensions, certain dimensions show greater predictive validity in the relationship between sleep and other dimensions of health (e.g., mental, physical, emotional, etc.). Those included in the B-SATED model, including sleep-related behaviors, satisfaction, daytime alertness, timing, efficiency, and duration, have historically shown strong associations to health outcomes (Hale et al., 2020; Meltzer et al., 2021). These domains often interact with one another and are shaped by socio-ecological factors (e.g., environment, family, school demands, individual traits, etc.) that may be overlooked when domains are considered in isolation (Meltzer et al., 2021). Figure 1 illustrates biopsychosocial factors which influence adolescent sleep across B-SATED outcome domains.

While sleep health is well-defined, and public health initiatives are beginning to advocate for improved sleep duration across age groups (American Heart Association, 2022), greater attention is needed to incorporate all relevant domains of sleep health, such as circadian sleep health, into clinical research and treatment of adolescent sleep health concerns.

## 2 Circadian sleep health

Sleep occurs within the body's overarching circadian pattern of activity and rest, which reflects a broader range of daily rhythmic tendencies in the body's neurophysiology and behaviors, involving endocrine, metabolic, immune, and other organ systems (Roenneberg and Merrow, 2016). Circadian sleep health, or the state of stability in daily endogenous patterns of activity and rest, is also strongly associated with a multitude of health outcomes (Baron and Reid, 2014; Fishbein et al., 2021; Sletten et al., 2020). Given the robust relationships between circadian rhythmicity and sleep (Meltzer et al., 2021), mental health (Bauducco et al., 2020; Duraccio et al., 2023; Merikanto et al., 2018), cognitive function (Meyer et al., 2022), and physical health (Logan et al., 2018; Scheer et al., 2009), sleep can be more fully understood as a subdomain within the broader framework of circadian health. With sleep's connection to broader 24-h processes in mind, operationalized circadian sleep dimensions can reveal complex interactions between environment, behavior, biology, and the body's natural rhythm of activity and rest, illuminating potential treatment targets.

We propose that investigating circadian sleep health factors when assessing adolescent sleep allows for a more precise evaluation of sleep patterns and more detailed exploration of their effects on intervention outcomes, overall sleep health, and adolescent wellbeing. Circadian sleep rhythm is typically measured through chronotype (a trait-like expression of circadian timing measured through reported preferences for active and restful behavior), circadian timing (timing of biological processes of activity and restfulness), and circadian alignment (the agreement between biological sleep timing and sleep timing behaviors; see Table 2). Interventions that address both sleep and circadian dimensions, such as the Transdiagnostic Sleep and Circadian Intervention (TSC), may be especially well-equipped to identify factors driving unsatisfactory sleep in clinical settings, such as poor sleep duration and quality (Dong et al., 2019).

**Table 2:** Circadian sleep health dimensions and definitions.

Dimension	Definition
Chronotype	Individual, biologically-informed timing of activity and rest during the circadian period; influenced by individual and environmental factors (Roenneberg, 2012; Taylor and Hasler, 2018). Often characterized by a perceived preference toward “eveningness” or “morningness” (Samson, 2021).
Circadian timing	Endogenous physiological processes that determine circadian rhythms (i.e., when the body is prepared to sleep vs. wake); involves the circadian pacemaker within the suprachiasmatic nuclei (SCN; Bonmati-Carrion et al., 2014).
Circadian alignment	How closely an individual's sleep behavior matches their biological circadian timing (Scheer et al., 2009).
Social jetlag	The discrepancy in timing of sleep between workdays/weekdays and free days/weekends; reflects misalignment between social and biological timing of sleep (Wittmann et al., 2006).
Sleep midpoint	Clock time of the middle point of sleep duration period (halfway between sleep onset and sleep offset; Morales-Ghinaglia and Fernandez-Mendoza, 2023).

This table highlights core dimensions but may not encapsulate all possible circadian dimensions that evolve as circadian sleep health science progresses.

Although research on circadian sleep health in adolescence is limited, establishing standardized assessment methods across research and clinical settings is critical for improving quality of care via greater concordance and generalizability of research findings. This paper serves as an introductory guide for early-career trainees, summarizing unique adolescent circadian contexts and providing validated measures for both research and clinical use by (1) identifying best-practice tools for defining and assessing circadian sleep health, (2) emphasizing a multimodal approach to assessment, and (3) illustrating how these methods can enhance intervention outcomes. Readers are assumed to have a basic understanding of circadian rhythmicity, research psychometrics, and factorial design.

## 3 Assessment of circadian sleep health

Due to their unique operationalizations, each core dimension of circadian sleep health, chronotype, circadian timing, and circadian alignment requires a distinct approach to measurement. Understanding how these dimensions differ from one another and identifying appropriate tools for their assessment are key to effectively diagnosing and treating circadian sleep health-related sleep disturbances in adolescents. The following sections provide detailed explanations of each circadian sleep health dimension and validated, best-practice measures for their assessment.

### 3.1 Chronotype

An individual's chronotype describes the biologically influenced preference in timing of daytime activities (i.e., when they feel most alert) and sleep behaviors (i.e., when they prefer to sleep) throughout the 24-h day (Bauducco et al., 2020). Typically, its operationalization includes timing of sleep behaviors, cognitive alertness, physical activity, and energy throughout the day (Wiłkość-Debczyńska and Liberacka-Dwojak, 2023). Chronotype is relatively stable but does shift with age, tending to be earlier in childhood, shifting later during adolescence and young adulthood, and returning to an earlier preference in later adulthood (Taylor and Hasler, 2018). Genetics also appear to play a large role in contributing to chronotype preference (Emmanuel and von Schantz, 2018; Kalmbach et al., 2017; Yamazaki and Goel, 2020), although trends show cross-cultural and geographical differences in chronotype (Emmanuel and von Schantz, 2018; Sultana, 2024).

Eveningness/morningness preference is a dimension of chronotype that provides information on individual's preferences for the timing of certain activities and their general alertness throughout the day. Within this dimension, chronotype is typically characterized by *eveningness* or *morningness*, or more colloquially *night owls* or *morning larks* (Samson, 2021), to help categorize nighttime and daytime behavioral pattern trends. For example, those with a preference toward the evening chronotype tend to wake up later in the day, have higher periods of alertness during the late afternoon and evening hours, and do not feel physiologically sleepy until the early morning hours. Alternatively, those who lean toward the morning chronotype tend to wake up earlier in the day, have higher periods of alertness during the morning hours, find it more difficult to maintain alertness and productivity in the afternoon hours, and feel physiologically sleepy in the evening. However, recent research suggests that the majority of individuals fall somewhere on a spectrum between true “morningness” and “eveningness” (Roenneberg, 2015; Roenneberg et al., 2007; Sultana, 2024), often referred to as “intermediate chronotype.” Colloquially, these have been referred to as *hummingbirds* (Sultana, 2024).

#### 3.1.1 Chronotype trends within adolescent populations

In general, evening chronotypes tend to have higher associations with negative health outcomes (Sempere-Rubio et al., 2022; Taylor and Hasler, 2018). In adolescent populations, these include increased risk and occurrence of insomnia, behavioral and emotional difficulties, lower academic performance, and attention deficits (Duraccio et al., 2023; Tokur-Kesgin and Kocoglu-Tanyer, 2021). Evening chronotype can also contribute to short sleep, as adolescents with evening chronotypes tend to have later timing of sleep onset (Lang et al., 2022), decreasing the amount of sleep that occurs before they are required to wake for school in the morning.

Interestingly, adolescents with an evening chronotype who are able to obtain longer sleep durations (i.e., sleep aligned with their chronotype) have exhibited lower risks of mood or anxiety disorders, obesity, and healthier physical and mental health across emotional, cognitive, and social dimensions, compared to adolescents with an evening chronotype that had shorter sleep duration (Dong et al., 2019). This suggests that social demands, such as early school start times, that conflict with adolescents' chronotypic tendencies toward later sleep schedules, could be major contributors to both inadequate sleep and associated negative health outcomes in this age group (Crowley et al., 2018; Tokur-Kesgin and Kocoglu-Tanyer, 2021). If modifiable social factors account for all or partial variance in health outcomes associated with chronotype, this construct could be viewed as amenable to change, rather than solely fixed. In this vein, some researchers suggest that light intervention therapies could be used to advance sleep onset and thereby increase sleep duration (Chesson et al., 1999) in addition to behavioral therapies that aim to slowly advance bedtime earlier in the evening (Harvey and Buysse, 2017; Harvey et al., 2021). As such, while a delay in chronotype is expected in adolescence, interventions that aim to pull chronotype earlier (thus facilitating opportunity for increased sleep duration) may help minimize the impact of a later chronotype on overall adolescent health.

#### 3.1.2 Assessment of chronotype within adolescent populations

Because chronotype is often expressed via a person's preference for timing for activity and rest and general trends of alertness throughout the day (Chauhan et al., 2023), self-report measures are the most generally agreed-upon method of assessing chronotype. Table 3 summarizes validated self-report measures of chronotype. Measures are organized to help early career trainees choose appropriate instruments based on participant age, purpose, constructs, and accessibility. DOI links for publicly available measures are provided in the References section.

**Table 3:** Established measures of chronotype.

Measure	Key citations	Items	Age	Primary purpose	Constructs assessed	Access
Morningness-Eveningness Questionnaire (MEQ)^*^	Horne and Ostberg, 1976; Shahid et al., 2012a; Smith et al., 1989; Thun et al., 2012	19	18–32	Standard self-report measure of adult chronotype; assesses preferred timing of daily alertness and activity to classify morning-evening preference.	• Morningness-eveningness preference • Timing of alertness and peak energy • Sleep-wake preferences • Preferred timing of daily activities (e.g., meals, exercise)	DOI
Reduced Morningness-Eveningness Questionnaire (rMEQ)	Adan and Almirall, 1991; Danielsson et al., 2019	5	17–50	Brief version of the MEQ designed for rapid assessment of adult chronotype with reduced participant burden.	• Sleep/wake preference • Perceived daily energy peak	DOI
Morningness-Eveningness Questionnaire for Children & Adolescents (MEQ-CA)	Tonetti et al., 2015	19	12–20	Developmentally adapted version of the MEQ for assessing chronotype in children and adolescents using school-relevant wording.	• Morningness-eveningness preference • Timing of alertness and peak energy • Sleep-wake preferences • Preferred timing of daily activities (e.g., meals, exercise)	Contact Dr. Tonetti
Reduced Morningness-Eveningness Questionnaire for Children and Adolescents (rMEQ-CA)	Paciello et al., 2022; Tonetti et al., 2024	5	13–16	Brief child/adolescent adaptation of the rMEQ for rapid assessment of chronotype. *At time of publication, English translation is available but not yet validated*.	• Sleep/wake preference • Perceived daily energy peak	Contact Dr. Tonetti
Morningness-Eveningness Stability Scale (MESC)	Carskadon et al., 1993; Tonetti et al., 2015	10	8–19	Child-specific measure of morningness-eveningness preference designed for school-aged populations.	• Preferred timing of activities (school tests, physical activity) • Sleep-wake timing • Energy and alertness	DOI
Munich Chronotype Questionnaire (MCTQ)^*^	Di Milia et al., 2013; Roenneberg et al., 2003; Shahid et al., 2012b; Zavada et al., 2005	19	18+	Detailed assessment of habitual sleep timing on workdays and free days that estimates chronotype and social jetlag from actual sleep behavior rather than preference.	• Sleep timing • Sleep midpoint • Social jetlag • Light exposure • Sleep latency • Self-reported chronotype	DOI
Composite Scale of Morningness (CSM)	Horne and Ostberg, 1976; Randler, 2009; Shahid et al., 2012c; Smith et al., 1989	13	9–20 (16+)	Alternative chronotype questionnaire adapted from the MEQ with greater emphasis on morningness while retaining assessment of eveningness.	• Morning affect • Morning alertness • Ease of waking • Sleep-wake preferences • Activity timing	DOI
Circadian Energy Scale (CIRENS)^*^	Ottoni et al., 2011, 2012	2	18+	Ultra-brief measure of diurnal energy patterns that can provide an indirect estimate of chronotype	• Morning energy • Evening energy • Afternoon energy (optional)	DOI
Sleep Timing Questionnaire (STQ)	Monk et al., 2003; Shahid et al., 2012d; Tremaine et al., 2010	18	11–16 OR 20–82	Questionnaire assessing habitual sleep schedules and sleep regularity; useful for estimating chronotype-related	• Habitual bedtime • Habitual wake time • Sleep regularity • Night awakenings	DOI
				sleep timing but not designed as a direct chronotype measure.		
Morningness-Eveningness Stability Scale, Improved (MESSi)	Faßl et al., 2019; Randler et al., 2016; Weidenauer et al., 2021	15	11–17 OR 18–76	Multidimensional chronotype questionnaire measuring morning affect, eveningness, and day-to-day stability of circadian functioning. Currently validated primarily in German populations.	• Morning affect • Eveningness • Stability/amplitude of daily rhythms	DOI
Children's Chronotype Questionnaire (CCTQ)	Jafar et al., 2017; Simpkin et al., 2014; Werner et al., 2009	27	4–11	Parent-report questionnaire assessing chronotype and habitual sleep timing in young children.	• Sleep timing • Sleep midpoint • Social jetlag • Activity timing • Parent-rated chronotype	DOI
School Sleep Habits Survey (SSHS)	Shahid et al., 2012e; Wolfson and Carskadon, 1998; Wolfson et al., 2003; Ziporyn et al., 2017	63	13–19	Comprehensive assessment of adolescent sleep habits and daytime functioning that includes several chronotype-relevant sleep timing variables.	• Sleep timing • Sleep regularity • Daytime sleepiness • Social jetlag • Academic functioning	DOI
Preferences Scale (PS)^*^	Smith et al., 2002, 1993	12	18–22	Brief preference-based questionnaire that estimates chronotype using preferred timing of daily activities rather than clock-time questions.	• Preferred timing of activities • Sleep-wake preferences • High-energy task preference	DOI

This table highlights commonly used established measures of chronotype but is not intended to be comprehensive and will continue to evolve as the field of circadian sleep health expands. References, including DOI links where available, are provided in the references list. Validation references are included where applicable; readers seeking detailed psychometric evidence should consult the cited publications. Measures marked with an asterisk (^*^) were originally developed for adults but are included because they are commonly used in research and clinical work with older adolescents and emerging adults. Researchers should consider the developmental appropriateness and available psychometric evidence when selecting adult-normed measures for adolescent samples.

#### 3.1.3 Recommendations for future methods of measuring chronotype

For future research involving chronotype, it would be valuable to explore the long-term health effects of chronotype advancement through interventions that target circadian timing, such as strategically timed light exposure and melatonin administration (e.g., TranS-C, phase advancement protocols). We also encourage clinicians and researchers to more comprehensively assess contextual factors, including light exposure, physical activity timing, and meal timing patterns when examining chronotype. Although these behaviors do not measure chronotype itself, they represent chronotype-relevant environmental and behavioral factors that may influence or reflect an individual's chronotype preference. For example, professionals might ask participants to answer questions such as, “I typically turn on the lights around X time,” “the brightest light I am exposed to each day occurs at X time,” or “I prefer to eat, work on school assignments, go to the gym, etc. at X time.” These contextual questions could help capture how an individual's chronotype relates to their daily behaviors and environment.

#### 3.1.4 Strengths and limitations of chronotype assessment approaches

Chronotype assessment methods vary in the extent to which they measure subjective preference vs. habitual rest-activity timing. Most validated chronotype measures rely on self-report questionnaires, which provide a practical and scalable approach for assessing individual differences in preferred timing of sleep, activity, alertness, and energy.

Preference-based questionnaires (e.g., MEQ-CA, rMEQ-CA, MESC, CSM, MESSi) represent the most used approach for assessing chronotype. These measures are brief, inexpensive, and easy to administer in research, school-based, and clinical settings, making them highly accessible for large-scale screening and longitudinal monitoring. Adolescent-specific measures, including the MEQ-CA, provide developmentally appropriate wording and content relevant to adolescent schedules and functioning (Faßl et al., 2019; Tonetti et al., 2015). However, questionnaire-based approaches primarily assess self-reported preference rather than directly observed sleep timing. Responses may be influenced by recall accuracy, self-awareness, and current environmental demands and may not fully capture actual behavioral patterns (Paciello et al., 2022; Tonetti et al., 2015). Additionally, although several adolescent chronotype measures demonstrate acceptable reliability and validity, differences in item content and scoring procedures limit direct comparability across instruments (Paciello et al., 2022). Briefer chronotype measures (e.g., rMEQ-CA and CIRENS) can increase feasibility of chronotype assessment in settings where assessment time is limited, though abbreviated measures in generally typically provide a narrower assessment and may provide less information regarding specific patterns of sleep timing, activity preference, and daily functioning compared with standard questionnaires (Danielsson et al., 2019; Ottoni et al., 2011).

Sleep timing-based questionnaires (e.g., MCTQ, STQ, and SSHS) provide an alternative approach to assessment by estimating chronotype from reported habitual sleep schedules rather than solely from subjective preference. These measures may provide clinically meaningful information regarding actual sleep timing, sleep regularity, and weekday-weekend stability in chronotype (Monk et al., 2003; Roenneberg et al., 2003; Wolfson and Carskadon, 1998). Given their focus on real-world sleep patterns, these approaches may be particularly useful for identifying behavioral targets for sleep interventions in clinical settings. However, reported sleep timing remains influenced by external constraints (e.g., school schedules, extracurricular activities, and family routines), and therefore may not fully represent intrinsic choronotype preference (Roenneberg et al., 2003; Saxvig et al., 2013). Additionally, longer questionnaires such as the SSHS may provide broader clinical information but require greater time and effort from respondents.

### 3.2 Circadian timing

Circadian timing is closely related to chronotype but is distinct enough to warrant separate examination. While chronotype reflects *biologically influenced* preferences for sleep/wake behavior and patterns associated with sleep timing, circadian timing refers to the *endogenous* rhythms of sleep and wakefulness, regulated by the suprachiasmatic nucleus (SCN) in the hypothalamus. Circadian timing is closely related to scheduled and perceived daily metabolic needs and, similar to chronotype, is also influenced by environmental cues such as light exposure, meal timing, and physical activity (Hand et al., 2023; Lewis et al., 2018, 2020). In other words, chronotype is the behavioral expression of an individual's sleep/wake preference, while circadian timing reflects underlying biological rhythms that likely contribute to that preference. Interventions aimed at adjusting circadian timing include strategically timed (typically morning) bright light therapies which tune the circadian clock via activation of the suprachiasmatic nucleus via ocular photoreceptors (Gooley, 2008) and early morning exercise (Lang et al., 2022). In certain cases, chronobiotic substances such as melatonin can help advance and entrain circadian timing, even at lower doses such as 0.1–0.5 mg (Cruz-Sanabria et al., 2023), though effectiveness in adolescent populations is mixed (Bartlett et al., 2013).

#### 3.2.1 Characteristics of circadian timing within adolescent populations

Research strongly suggests that during puberty and adolescence, a shift in circadian timing leads to later sleep onset (Crowley et al., 2014). This shift is driven primarily by a natural delay in melatonin release, which has been observed independent of evening artificial light exposure (Crowley et al., 2014), suggesting that adolescent physiology naturally shifts circadian timing later. Although this delay is initiated innately, external behaviors (e.g., caffeine consumption, alcohol consumption, jobs, homework, sports, poor time management, social engagement, etc.) can further delay the release of melatonin, thus disrupting the body's preferred rest period and decreasing overall sleep quality and duration (Reichert et al., 2021). As such, like chronotype, circadian timing can be influenced by a complex interplay between biological, social, and environmental factors.

Later melatonin onset is associated with higher rates of negative health outcomes. In adolescent populations, those with later melatonin onset tend to endorse higher levels of depression (Duraccio et al., 2023) and possess higher BMI scores (Duraccio et al., 2024b) than peers with earlier melatonin onset. Later melatonin onset is also linked to negative adolescent mood patterns, including more negative evening affect and less positive affect (Dolsen and Harvey, 2018). Biological delays in melatonin secretion during adolescence shift sleep onset later and reduce sleep duration in those with early social demands, similar to patterns observed in evening chronotypes, posing risks to adolescent sleep and circadian sleep health.

#### 3.2.2 Assessment of circadian timing

Circadian timing is typically assessed using physiological markers, such as melatonin, temperature, or cortisol, though behavioral patterns from actigraphy can also provide indirect insight. Validated methodologies are presented in Table 4.

**Table 4:** Established measures of circadian timing.

Measure	Key citations	Primary purpose	Procedural overview
Salivary dim-light melatonin onset (DLMO; in-laboratory)	Crowley et al., 2016; Kennaway, 2023; Voultsios et al., 1997	Measures the timing and magnitude of pre-bedtime melatonin secretion to estimate endogenous circadian phase. Salivary DLMO is typically defined when salivary melatonin exceeds 4 pg/mL. Salivary DLMO is validated among adolescent populations.	In a dimly lit room (< 20 lux), saliva samples are collected every 30–60 min beginning 3–5 h before habitual bedtime and continuing until 3–6 h afterward.
Salivary dim-light melatonin onset (DLMO; in-home)	Burgess et al., 2015; Murray et al., 2024; Pullman et al., 2012; Saxvig et al., 2013	Measures circadian phase using the same principles as in-laboratory DLMO while allowing assessment in a naturalistic environment. Preliminary evidence supports validity in adolescents, although additional psychometric evaluation is needed.	Participants remain in dim light from 7 h prior to habitual bedtime until 1 h after. Saliva samples are self-collected every hour, frozen, and returned to the laboratory. Low lux lighting and light-blocking glasses are typically provided.
Plasma-based dim-light melatonin onset (DLMO; in-laboratory)	Dermanowski et al., 2022; Kennaway, 2019; Ozkan et al., 2012	More resource-intensive than salivary DLMO but provides a detailed profile of melatonin secretion across the circadian cycle. Melatonin is quantified using immunoassay or high-performance liquid chromatography (HPLC). No published validation studies in adolescents were identified.	Plasma samples are collected repeatedly over an 18–24-h period via an indwelling intravenous catheters to characterize the full melatonin rhythm.
Sleep midpoint	Wirz-Justice, 2007; de Souza and Hidalgo, 2015; Lovato et al., 2016	Repeated assessment of sleep midpoint estimates preferred sleep timing to be calculated and serves as an indirect marker of circadian phase. Accuracy is reduced by irregular or disrupted sleep schedules.	Sleep midpoint is calculated from sleep onset and offset (via actigraphy or sleep diary) through actigraphy software or manually by dividing the total sleep duration in half and adding that time to the time of sleep onset.
Core body temperature	Murphy and Campbell, 1997; Szymusiak, 2018; Waterhouse et al., 2005	The timing of the core body temperature nadir (lowest point) provides an estimate of circadian phase.	Core body temperature is continuously monitored (typically via rectal probe), and the timing of the temperature nadir is identified relative to sleep period and/or melatonin rhythm.
Actigraphy	Ancoli-Israel et al., 2003; Lockley et al., 1999; Lynch et al., 2019; Marino et al., 2013; Scott et al., 2017	Measure rest-activity patterns over multiple days to estimate circadian timing and preferred sleep/activity schedules. Validated among adolescent populations.	Wrist- and waist-worn activity monitors are worn continuously (typically ≥7 days) to record activity and rest patterns, including habitual sleep timing.
Cortisol rhythmicity	Bailey and Heitkemper, 2001; Castro et al., 2000; O'Byrne et al., 2021	Diurnal cortisol secretion patterns provide an indirect marker of circadian timing and biological arousal rhythms.	Cortisol concentrations are measured repeatedly over a 24-h period using blood or saliva samples to characterize the daily secretion rhythm.
DLMO calculator	Cheng et al., 2021	Using mathematical models and actigraphy data to estimate DLMO timing as a proxy for circadian phase. Developed by Drs. Philip Cheng and Olivia Walch.	Actigraphy data (.csv) are uploaded to the online calculator, which estimates the timing of DLMO. Access is available via https://www.predictdlmo.com/.
Transcriptomic biomarkers	Laing et al., 2017	Circadian phase is estimated from rhythmic gene expression patterns in blood. This method has not yet been validated in adolescents.	Blood samples are analyzed for circadian-related mRNA transcriptome expression in the bloodstream to estimate melatonin phase and circadian timing.

This table highlights commonly used measures of circadian timing but is not intended to be comprehensive. As the field of circadian sleep health evolves, additional measures and supporting evidence may emerge; therefore, emerging assessment methods are included alongside more established approaches in the current table. References, including DOI links where available, are provided in the references list. Validation references are included where applicable; readers seeking detailed psychometric evidence should consult the cited publications.

#### 3.2.3 Recommendations for future methods of measuring circadian timing

Future research into circadian timing could benefit from expanded hormonal measures related to circadian timing and rhythm. This could include more continuous measurement of melatonin levels throughout the day (vs. exclusive focus on hours surrounding bedtime, as is the current standard) that take place at home to provide a more complete picture of circadian phase shifts and daily trends. Beyond melatonin, researchers should continue to validate other biological markers in adolescents, such as cortisol rhythms and temperature within the context of sleep and circadian timing to better understand trends in energy and metabolism (Challet, 2015). These trends may inform functional analyses of daytime sleepiness or illuminate endogenous circadian patterns which may be obscured by exogenous sleep behaviors (such as light exposure habits, by which brief measures of melatonin levels can be influenced). Further exploration could include other hormones related to broader circadian rhythmicity, such as leptin or growth hormone, to see how these interplay with sleep and the feasibility of their measurement as a measure of circadian timing (Kim et al., 2015). Doing so could foster a more comprehensive understanding of an individual's circadian hormonal functioning with respect to their circadian timing. Finally, additional work is needed to validate less-intensive methods of estimating circadian timing (including actigraphy, DLMO prediction algorithms) to help broaden the reach of circadian timing as an outcome in research.

#### 3.2.4 Strengths and limitations of circadian timing assessment approaches

Direct physiological measures (e.g., salivary or plasma DLMO, core body temperature rhythms, cortisol rhythmicity, emerging transcriptomic biomarkers) provide the most precise assessment of circadian phase but require greater participant burden, specialized equipment, and financial resources. Salivary-based DLMO is considered one of the most established measures of endogenous circadian phase and is widely used as a standard reference for assessing circadian timing (Crowley et al., 2016; Saxvig et al., 2013). Laboratory collection protocols require several hours of evening sampling, specialized laboratory facilities, trained personnel, and substantial participant effort, limiting feasibility for routine clinical use (Kennaway, 2023). Home-based salivary DLMO protocols have emerged to improve accessibility by allowing participants to collect samples in naturalistic settings to reduce participant burden. However, home-based protocols place greater procedural burden on the participant (e.g., adherence to dim-light conditions, accurate sample collection, appropriate sample management, etc.) which may limit feasibility (Burgess et al., 2015; Murray et al., 2024; Saxvig et al., 2013). Plasma-based melatonin assessment, cortisol rhythmicity, and emerging transcriptomic gene expression monitoring provide the most detailed characterization of circadian timing and may offer advantages when comprehensive physiological profiling is required, but it is substantially more invasive and resource intensive than salivary measures (Kennaway, 2019). These limitations make these approaches impractical for routine clinical use and large-scale adolescent research, though they may serve specific research or specialized clinical purposes.

Conversely, behavioral approaches to estimating circadian timing (e.g., sleep midpoint, actigraphy-derived sleep timing or DLMO prediction models) provide more accessible and scalable alternatives by estimating circadian timing from habitual sleep-wake patterns. These methods are particularly useful for clinical and population-based applications because they allow assessment across multiple days in naturalistic environments with lower participant burden and cost. Actigraphy and sleep timing measures can characterize habitual sleep schedules, variability, and behavioral patterns relevant to circadian alignment and intervention planning (Lynch et al., 2019; Scott et al., 2017). However, behavioral measures reflect the interaction between biological timing and external influences (e.g., school schedules, social demands, light exposure) and therefore should be interpreted as estimates or behavioral circadian timing rather than direct measures of endogenous circadian phase.

### 3.3 Circadian alignment

Circadian alignment refers to how closely an individual's sleep behavior matches their biological circadian timing (Scheer et al., 2009). When the difference between a person's circadian timing and sleep behavior becomes sufficiently incongruent (or mismatched), it is referred to as circadian *mis*alignment (Baron and Reid, 2014). At clinical levels, this misalignment can meet diagnostic criteria for a range of circadian rhythm sleep disorders (CRSDs), where a person's internal sleep/wake cycle is skewed by environmental factors such as work, school, social responsibilities, or light exposure (Zhu and Zee, 2012). Common CRSDs include Delayed Sleep Phase Disorder, Advanced Sleep Phase Disorder, Irregular Sleep Wake Rhythm, and Free-Running Disorder (Yanagihara et al., 2014; Zhu and Zee, 2012). Chronic maintenance of this misalignment is associated with increased risk of weight gain, hypertension, cardiovascular disease (Zhu and Zee, 2012), mood dysregulation, and changes in metabolism and behavior (Baron and Reid, 2014; Duraccio et al., 2024a). As circadian alignment is well connected to a person's physical, mental, and emotional health, it can serve as a powerful intervention target. Given that circadian misalignment reflects incongruence between an individual's biologically preferred sleep timing and actual sleep behavior, interventions tend to revolve around behavioral changes. For example, the Transdiagnostic Intervention for Sleep and Circadian Dysfunction (TSC) incorporates circadian-focused strategies like slowly advancing sleep schedules, increasing energy expenditure in the daytime (dual process model), providing psychoeducation and motivational interviewing around sleep health behaviors, and utilizing strategic light exposure to realign biological rhythms and desired sleep-wake patterns (Harvey and Buysse, 2017; Harvey et al., 2021).

#### 3.3.1 Characteristics of circadian misalignment within adolescent populations

Due to the combination of biopsychosocial factors commonly present during the adolescent developmental period (later chronotype (Baron and Reid, 2014), biological shifts toward later sleep onset (Kirby et al., 2011), incongruent school start times and social schedules, etc.), adolescents are more prone to circadian misalignment, which increases their susceptibility to adverse health consequences related to this condition (Crowley et al., 2018).

At clinical levels, Delayed Sleep Phase Disorder is one of the more prevalent CRSDs, with a prevalence rate of about 0.2–16% within general adolescent cohorts (Gradisar and Crowley, 2013; Kansagra, 2020; Micic et al., 2016). Even subclinical levels of circadian misalignment appear to have an impact on metabolism (Morales-Ghinaglia et al., 2024) and can increase the general risk of disease (Kelly et al., 2018), specifically raising the risk of cardiovascular disease (Fishbein et al., 2021) in both adult and adolescent populations.

#### 3.3.2 Assessment of circadian alignment

Assessment of circadian alignment relies on a combination of subjective self-report measures or behavioral sleep information (to ascertain sleep and wake schedules) and objective biomarkers. Specifically, measurement of melatonin secretion is a common anchor for comparisons between endogenous sleep/wake preference (when sleep onset would naturally begin) and sleep/wake behavior observed via actigraphy. Validated measures are presented in Table 5.

**Table 5:** Established measures of circadian alignment.

Measure	Key citations	Primary purpose	Procedural overview
Social jet lag (SJL)	Duraccio et al., 2023; Henderson et al., 2019; Jankowski, 2017	Estimates misalignment between biological and social schedules by quantifying differences in sleep timing between work/school days and free days. Greater differences in sleep midpoint indicate greater circadian misalignment. Two common methods for calculating social jetlag are listed. Roenneberg's traditional method utilizes sleep midpoint to calculate social jetlag, while Jankowski's method utilizes either sleep onset or sleep-corrected sleep midpoints (see Jankowski, 2017 for additional detail).	**Roenneberg method:** 1. Calculate sleep duration on workdays *SD_*work*_ = wake time_*work*_ – sleep time_*work*_* 2. Calculate sleep duration on free days *SD_*free*_ = wake time_*free*_ – sleep time_*free*_* 3. Calculate the midpoint of sleep on free days *MSF = sleep time_*free*_ + (SD_*free*_/2)* 4. Calculate the midpoint of sleep on workdays *MSW = sleep time_*work*_ + (SD_*work*_/2)* 5. Compute social jetlag *(|MSF – MSW|)* **Jankowski method:** 1. Calculate sleep onset on free days 2. Calculate sleep onset on workdays 3. Calculate social jetlag (sleep-corrected) *SJ_*sc*_ =|sleep onset_*free*_ – sleep onset_*work*_|*
Circadian misalignment index (CMI) or phase angle	Fischer et al., 2016	Quantified alignment between endogenous circadian phase (DLMO) and behavioral sleep timing. Larger phase angles indicate greater separation, or misalignment, between biological melatonin timing and behavioral sleep patterns.	Calculated as the interval between melatonin onset and sleep onset or midpoint of sleep by subtracting the time of sleep onset or midpoint from time of melatonin onset. Sleep timing variables are typically derived from actigraphy or self-reported sleep diaries.
Composite phase deviation (CPD)	Fischer et al., 2016	Estimates misalignment activity-rest rhythms and environmental light-dark cycles. Not currently validated in adolescents.	Calculated using deviations between an individual's sleep midpoint and chronotype, as well as changes in sleep midpoint across consecutive days.
Inter-daily stability (IS)	Gillett et al., 2021; Zuurbier et al., 2015	Measures the consistency of rest-activity rhythms across days and the degree of alignment with the 24-h light-dark cycle. From this, degree of circadian alignment or disruption can be inferred. Validated in adolescent populations.	Calculated from multi-day actigraphy data as the ratio of variance in average daily patterns (e.g., hourly) to total activity variance across the recording period.

This table highlights commonly used measures of circadian alignment but is not intended to be comprehensive. As the field of circadian sleep health evolves, additional measures and supporting evidence may emerge; therefore, emerging assessment methods are included alongside more established approaches in the current table. References, including DOI links where available, are provided in the references list. Validation references are included where applicable; readers seeking detailed psychometric evidence should consult the cited publications.

#### 3.3.3 Recommendations for future methods of measuring circadian alignment

Assessing a combination of sleep behaviors *and* biomarkers, such as melatonin, cortisol, and glucose, can enhance both individual and provider understanding of a person's circadian profile, thereby informing more targeted interventions for circadian misalignment. Self-report measures that include light exposure, social/work schedules, and timing preferences may also inform assessment of circadian alignment when combined with biological measures that identify the timing of sleep phases and pressures within an individual. Furthermore, creating and validating algorithms to calculate deviations in sleep regularity and rhythmicity using wearable devices within adolescents will be a critical next step (Kim et al., 2022).

#### 3.3.4 Strengths and limitations of circadian alignment assessment approaches

Behavioral measures of circadian alignment (e.g., social jet lag, sleep midpoint differences, and inter-daily stability) provide accessible and scalable approaches for assessing alignment between habitual sleep-wake patterns and social schedules. Social jet lag, calculated from differences in sleep timing between school/workdays and free days, is particularly feasible given that it can be derived from sleep diaries, questionnaires, or actigraphy data and does not require specialized equipment (Duraccio et al., 2023; Jankowski, 2017; Roenneberg et al., 2019). Similarly, inter-daily stability derived from actigraphy provides information regarding the consistency of rest-activity rhythms and alignment with environmental light-dark cycles (Gonçalves et al., 2014; Smith et al., 2018). These approaches are well-suited for large-scale research and clinical monitoring of behavioral patterns that may contribute to circadian disruption. However, behavioral alignment measures cannot distinguish whether misalignment reflects delayed endogenous circadian timing, insufficient sleep opportunity, irregular schedules, or environment constraints (Crowley et al., 2014; Hasler et al., 2025), information which may be clarified via clinical interview.

CMI provides a flexible approach by allowing assessment of alignment using behavioral measures alone (e.g., actigraphy-derived sleep timing and chronotype) or by incorporating physiological markers of endogenous circadian timing (e.g., actigraphy-derived sleep timing and DLMO). This flexibility allows CMI to be adapted to different research and clinical contexts, although physiological approaches provide greater specificity regarding biological circadian misalignment.

## 4 Measurement of other factors that influence circadian sleep health

**Figure 2:**
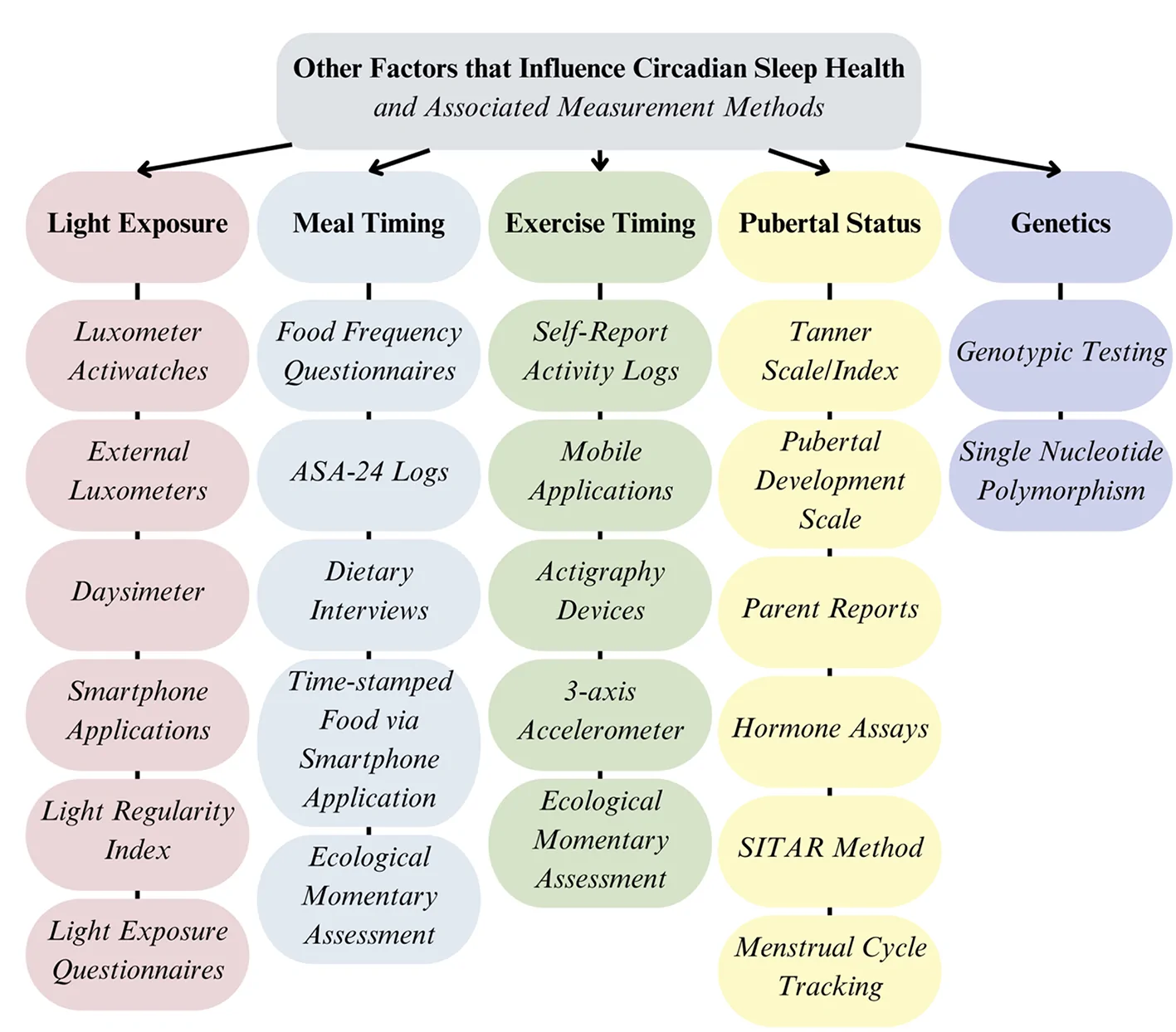
Other factors that influence circadian sleep health.

In addition to age-related biological factors, various internal and external factors appear to influence circadian sleep health (see Figure 2). These factors reflect the dynamic interaction between the body's intrinsic clock and environmental cues within circadian sleep health. Measuring these influences can support the development of sleep interventions that employ a multimodal approach to both assessment and treatment. These internal and external influences are briefly summarized below.

### 4.1 Light exposure

#### 4.1.1 Effects of light exposure on circadian sleep health

Environmental light functions as one of the strongest zeitgebers, or time cues, to synchronize the body's internal circadian rhythm with the solar day. Light influences the circadian system through specialized retinal pathways that send information about environmental light to the SCN, an area of the brain that promotes wakefulness and inhibits melatonin release. Light exposure works through these systems and can either advance (pull earlier) or delay (push later) circadian timing, depending largely on when it occurs (Hand et al., 2023; Meyer et al., 2022). Crowley and Eastman (2017) demonstrated that in late- to post-pubertal adolescents, bright light produces circadian shifts in a predictable, time-dependent pattern: light exposure during the early biological night (around bedtime) tends to delay circadian phase, where light exposure during the latter half of the biological night and early morning tends to advance circadian phase. Notably, the time at which light transitioned from delaying to advancing circadian phase occurred near the midpoint of sleep, suggesting that midpoint of sleep may help provide a practical anchor to guide light exposure as a circadian phase adjustment tool.

Beyond the timing of light exposure, the effects of light on circadian health also depend on its intensity, wavelength or spectral composition, duration, and the individual's sensitivity to light (Brainard et al., 2008; Gradisar et al., 2011; Kim and Casement, 2024; Meyer et al., 2022; Mireku et al., 2019; Misiunaite et al., 2020; Richardson et al., 2018; Ricketts et al., 2022). For instance, among adolescents with late bedtimes and short school-night sleep, 2.5 h of weekend morning bright light timed relative to the midpoint of weekend sleep produced a larger advance in DLMO than either dim room light or a shorter bright-light exposure, illustrating how timing, duration, and intensity interact to influence circadian phase (Misiunaite et al., 2020). Individual responsiveness to light also appears to vary across adolescence. For example, early- to mid-pubertal adolescents exhibit greater evening light-induced melatonin suppression than late- to post-pubertal adolescents, suggesting that developmental stage may influence sensitivity to the circadian effects of light (Crowley et al., 2015).

#### 4.1.2 Methods for measuring light

Light is most commonly measured in units of “lux,” or degree of intensity on a surface (Spitschan et al., 2019). Wrist-worn actigraphs with luxometers have historically been used to measure daily light exposure, though discontinuations of certain devices (i.e., Actiwatches) have prompted sleep researchers to seek alternative methods (van Duijnhoven et al., 2024). Validated but more obtrusive options include neck- or shoulder-worn external luxometers, such as the Daysimeter (Figueiro et al., 2011, 2013) or OcuLux (Vieira Dias et al., 2017). Smartphone applications provide a convenient option but have lower accuracy and reliability when compared to standardized luxometers (Kim et al., 2017; Melo et al., 2018). Deriving daily patterns of light exposure through actigraphy and applications can facilitate the calculation of a Light Regularity Index (LRI), which quantifies the consistency of a person's daily light exposure and is linked to regular sleep patterns (Hand et al., 2023). In absence of access to luxometer equipment, some self-reported measures, such as the Harvard Light Exposure Assessment (Bajaj et al., 2011), have been developed, but have not yet been psychometrically validated in adolescent populations.

### 4.2 Meal timing

#### 4.2.1 Effect of meal timing on circadian sleep health

The body's metabolism follows a diurnal rhythm, cycling between activity and rest (Huang et al., 2011; Peng et al., 2022). Meal timing serves as a non-photic zeitgeber (or a zeitgeber not related to light), anchoring circadian rhythms, reflecting individual circadian preferences, and influencing sleep (Wehrens et al., 2017). However, unlike light, which primarily entrains the SCN, meal timing exerts strong effects on peripheral oscillators (or localized circadian “clocks” throughout the body) located in metabolic tissues, such as the liver (BaHammam and Pirzada, 2023; Damiola et al., 2000; Reytor-González et al., 2025; Stokkan et al., 2001). Experimental evidence demonstrates that poor chrononutrition (e.g., eating at times that are irregular, unusually late, or out of sync with the body's natural circadian rhythm) can disrupt coordination between the brain's central clocks and the clocks in other organs and tissues (Damiola et al., 2000; Dibner et al., 2010; Pickel and Sung, 2020). More specifically, when the body's metabolic clock falls out of sync with the SCN, melatonin release may be delayed and cortisol levels may remain higher at night (Paragliola et al., 2025), contributing to later sleep timing and shorter sleep duration in adolescents and adults (Spaeth et al., 2019). On the contrary, adaptive chrononutrition (e.g., eating at times that are regular and earlier in the day) may help to synchronize peripheral clocks (like in metabolic organs and tissues) with the SCN. Human experimental research indicates that meal timing can act as a direct timing cue for these peripheral clocks in young adults; specifically, delaying meals can shift daily metabolic rhythms, even without observed changes in the SCN (Kessler and Pivovarova-Ramich, 2019; Wehrens et al., 2017) As such, assessing adolescent meal timing in conjunction with behavioral and/or physiological measurements of circadian timing may provide valuable insight into circadian alignment, risk for sleep and metabolic dysregulation, and potential metabolic contributors to sleep or circadian disturbance.

#### 4.2.2 Methods for measuring meal timing

Common measures of meal timing include the Food Frequency Questionnaires (Ambrosini et al., 2009; Matthys et al., 2007; Tabacchi et al., 2014) and the Automated Self-Administered 24-h (ASA24) Dietary Assessment Tool (Hughes et al., 2017). Dietary interviews allow detailed nutritional insight but may be less reliable in younger populations due to recall variability and desirability bias (Sharman et al., 2016). Smartphone apps can also be used to time stamp when food is ingested (Samad et al., 2022), while ecological momentary assessments reduce self-report bias by capturing real-time behaviors via third-party observation (Chiang and Lam, 2020). To create estimates of meal-circadian alignment (understanding when a person eats relative to their endogenous circadian phase), one can estimate a meal-to-DLMO phase angle, or the interval between an eating time and DLMO (zero would indicate eating at one's DLMO); eating the final meal closer to DLMO may indicate greater meal-circadian misalignment (Culnan et al., 2021). If caloric content is assessed, one can also estimate caloric-circadian alignment; caloric midpoint is first calculated as the time in which the participant has consumed 50% of that day's calories, and DLMO is then subtracted from the timing of caloric midpoint (McHill et al., 2017).

### 4.3 Exercise timing

#### 4.3.1 Effect of exercise timing on circadian sleep health

Exercise timing also serves as a zeitgeber for adolescent circadian rhythms, influencing heart rate, body temperature, skeletal muscle, and lung biology (Wolff and Esser, 2019). As an important non-photic zeitgeber, exercise can regulate the body's internal rhythms and may represent a behavioral intervention for circadian rhythm disorders (Richardson et al., 2017). Although exercise timing does not appear to alter chronotype itself, it can influence circadian alignment by delaying melatonin release, depending on the timing of activity (Buxton et al., 2003; Lewis et al., 2018). Similar to light exposure, physical activity appears to exhibit a phase response curve in which exercise performed during the biological morning can advance circadian timing, whereas exercise performed during the biological evening may delay circadian phase and melatonin onset (Thomas et al., 2020; Youngstedt et al., 2019, 2016). Exercise performed during the middle of the biological day appears to produce comparatively smaller phase-shifting effects (Youngstedt et al., 2019).

These findings suggest that exercise may serve as a useful non-photic entraining signal for adolescents, particularly for those with delayed sleep timing or behavioral circadian misalignment. In one of the first experimental studies to examine this in adolescents, Lang et al. (2022) randomized physically inactive adolescent males with a late chronotype to either a low-intensity morning activity (45 min of treadmill walking) or a sedentary activity condition. For 5 days, participants completed these conditions under dim-light conditions, and wake time/activity time advanced 30 min each day. Adolescents assigned to the morning condition demonstrated a 27.5-min DLMO advance (those in the sedentary condition had a 34.3 min delay). While, appropriately timed morning exercise paired with advancing wake time may advance circadian phase in adolescents (Lang et al., 2022), exercise later in the day could theoretically reinforce delayed timing, particularly when it is vigorous or occurs close to bedtime. Evidence regarding evening exercise remains mixed, with effects likely varying according to exercise intensity and duration, baseline sleep regularity, individual chronotype, and the interval between exercise and bedtime (Buman et al., 2014; Kim et al., 2023; Saidi et al., 2020; Stutz et al., 2019).

#### 4.3.2 Methods for measuring exercise timing

Exercise timing is commonly measured through retrospective questionnaires, self-reported logs, or mobile applications, where participants are asked to report the timing, during, intensity, and type of exercise; however, reliability varies by exercise type (Caputo et al., 2022; Silva et al., 2020) and can be subject to recall error or inaccurate perceptions of duration and/or intensity (Caputo et al., 2022; Silva et al., 2020). Ecological momentary assessments can help to reduce these recall errors by prompting participants to report exercise close to when it occurs in real time (Chiang and Lam, 2020). Wearable accelerometers provide more continuous, time-stamped measurements of movement for extended periods, offering more valid and detailed information on exercise timing and intensity (Chiang and Lam, 2020; Konstabel et al., 2019; Skatrud-Mickelson et al., 2011). However, accelerometers measure movement rather than intentional exercise, their estimates depend on where the device is placed and algorithms used to classify activity (Migueles et al., 2017), and they may also underestimate some activities like resistance training or cycling (Cerin et al., 2016; Herman Hansen et al., 2014). Combining accelerometry with exercise logs or ecological momentary assessment may help distinguish intentional exercise from other movements and provide context to help identify specific activity sessions (Dunton, 2017; Liao et al., 2015). When the research question concerns circadian misalignment, expressing exercise timing relative to sleep timing or, preferably, a biological marker of circadian phase (i.e., DLMO) may provide a more meaningful estimate of when exercise occurs within the individual's circadian cycle (Pandi-Perumal et al., 2007; Thomas et al., 2020).

### 4.4 Pubertal status

#### 4.4.1 Effect of pubertal status on circadian sleep health

Sexual maturation involves various hormonal and developmental changes that affect circadian sleep health, shifting chronotype toward eveningness, endogenous circadian timing toward a later phase, and sleep onset toward later times (Hagenauer and Lee, 2012). These shifts do not appear to be explained entirely by adolescent behavior, as adolescents who are more pubertally mature have later DLMO than pre-pubertal adolescents, even when maintaining similar sleep-wake schedules (Taylor et al., 2005). Pubertal development is also associated with slower accumulation of homeostatic sleep pressure, which may allow adolescents to remain awake longer into the biological evening and contribute to later sleep onset (Jenni et al., 2005). Early pubertal development may also be associated with increased light sensitivity to evening light, elevating the risk of melatonin suppression and delayed sleep onset delay due to evening light exposure (Crowley et al., 2015) though findings are mixed (Hartstein et al., 2024; Nagare et al., 2019). As circadian timing shifts later while school and social schedules continue to require early morning wake times, adolescents may experience greater misalignment between their biological rhythms and externally imposed schedules (Crowley et al., 2018). Given the strong correlations between puberty and circadian sleep health, measuring pubertal status is crucial to understanding adolescent sleep disruptions and normative circadian shifts as well as the adolescent vulnerability to circadian misalignment.

#### 4.4.2 Methods for measuring pubertal status

Pubertal status can be measured through both self-report and physiological measures. The Tanner (Emmanuel and Bokor, 2022) and Pubertal Development (Koopman-Verhoeff et al., 2020) Scales both assess physical changes and sexual maturity (Bond et al., 2006). These sexual maturation questionnaires can be completed via adolescent self-report or parent report, both of which have been validated as reliable measures of pubertal status (Koopman-Verhoeff et al., 2020). Hormone assays, which track hormonal shifts (Dorn and Biro, 2011), and the Superimposition by Translation and Rotation (SITAR) method, which uses growth curve modeling to measure height changes over time (Cole et al., 2010), allow for physiological measurement of pubertal status. Additionally, the American Academy of Pediatrics recommends tracking onset of menarche and menstrual cycling as a vital tool in assessment of developmental status in biological females (Diaz et al., 2006).

### 4.5 Genetics

#### 4.5.1 Effect of genetics on circadian timing

Several genetic factors have been associated with both circadian rhythm regulation and chronotype preferences. The PER3 gene contributes to circadian timing, with longer variations of this gene (e.g., PER3^5^ allele and PER3^5/5^ genotype) are generally associated with earlier chronotype or earlier sleep timing, whereas shorter variations (PER3^4^) has been linked to later chronotype and/or delayed sleep phase (Archer et al., 2003; Hida et al., 2014; Jones et al., 2007). Variations in the CLOCK gene, which is involved in regulating daily rhythms, can influence diurnal preference, with the T allele associated with more morning lark patterns and the C allele more night owl patterns, though these findings need replicated in adolescents (Mishima et al., 2005; Pedrazzoli et al., 2010). It is more likely that chronotype is a polygenic trait that is influenced by many genetic variants; these genetic factors may influence duration of the circadian period, sensitivity to light, and the timing of melatonin secretion, thereby influencing an individual's preferred timing for sleep and wakefulness (Chellappa, 2021; Jagannath et al., 2017; Liu et al., 2022). Emergent adolescent research suggests that these genetic factors may become more strongly associated with chronotype and sleep timing across development, particularly as puberty progresses (Hernandez et al., 2025; Merikanto et al., 2018).

#### 4.5.2 Methods for measuring genetics

Understanding an individual's genetic background requires genomic measures, such as targeted genotyping, DNA sequencing, or single nucleotide polymorphism (SNP) analysis (Gottlieb et al., 2007; Lane et al., 2023). SNP analysis allows researchers to evaluate specific genetic variants linked with sleep disorders, chronotype, or circadian timing (Gottlieb et al., 2007; Lane et al., 2023; Baris et al., 2023; Ferguson et al., 2018). However, genetic testing reflects susceptibility rather than certainty, as these genetic influences interact with age, environment, and behavior.

## 5 Measurement of circadian sleep health factors via consumer wearables

Consumer wearable devices (e.g., Fitbit, Garmin, Oura Ring, Apple Watch, etc.) have become increasingly common among adolescents and represent a scalable, low-burden method for longitudinal assessment of sleep-wake behavior in naturalistic settings (Dunn et al., 2018). Although prevalence varies across populations, their growing use introduces important opportunities for integrating longitudinal behavioral sleep monitoring into circadian sleep health research and clinical work alongside traditional clinical and research-grade tools (Piwek et al., 2016).

Consumer wearables provide ecologically relevant estimates of behavioral sleep patterns, including sleep timing, duration, and variability (Baumert et al., 2022; Chinoy et al., 2022). However, their validity is constrained by their underlying measurement approach (Evenson et al., 2015). Most devices infer sleep from peripheral physiological and behavioral signals (e.g., movement, heart rate, and in some devices, temperature or heart rate variability) rather than directly measuring endogenous circadian phase or sleep architecture (De Zambotti et al., 2019). As a result, evidence from validation studies indicates that consumer wearables perform moderately comparably to research-grade actigraphy for estimating sleep duration and timing (Chee et al., 2021; Chinoy et al., 2022; Lee et al., 2019). However, they exhibit reduced accuracy in sleep staging and wake detection, particularly in adolescents, for whom polysomnography (PSG) remains the gold standard (Chee et al., 2021; Lee et al., 2019). Accordingly, outputs from consumer wearables should be interpreted as estimates of behavioral sleep-wake patterns rather than as direct measures of physiological circadian timing (Lau et al., 2022).

While research in young adolescents is limited, behavioral sleep-wake pattern estimates from consumer wearables appear particularly well-suited for assessing derived behavioral chronotype indicators (e.g., timing of sleep-wake activity) and circadian alignment indicators (e.g., social jetlag, sleep midpoint, variability in sleep timing, regularity of sleep-wake patterns) in emerging adult and adult samples (Cooper et al., 2026; Fudolig et al., 2025). In contrast, they are not well-suited for estimating endogenous circadian phase (e.g., dim light melatonin onset) or detailed sleep architecture.

Incorporating consumer wearable technology into clinical and research contexts aligns with the National Sleep Foundation's consensus recommendations, which emphasize the responsible integration of consumer sleep technologies into sleep health monitoring while maintaining appropriate standards for validity, transparency, and clinical interpretation (Dzierzewski et al., 2026). Importantly, current technological development of wearable devices and proprietary sleep scoring algorithms continues to outpace validation science. As such, there is substantial variability across devices in the definition, processing, and reporting of sleep metrics, limiting cross-device comparability and interpretability of findings (Baumert et al., 2022; De Zambotti et al., 2019). Despite these limitations, consumer wearables provide valuable complementary information to established clinical and research-grade methods, including PSG and actigraphy.

## 6 A multimodal approach to measuring circadian sleep health

**Figure 3:**
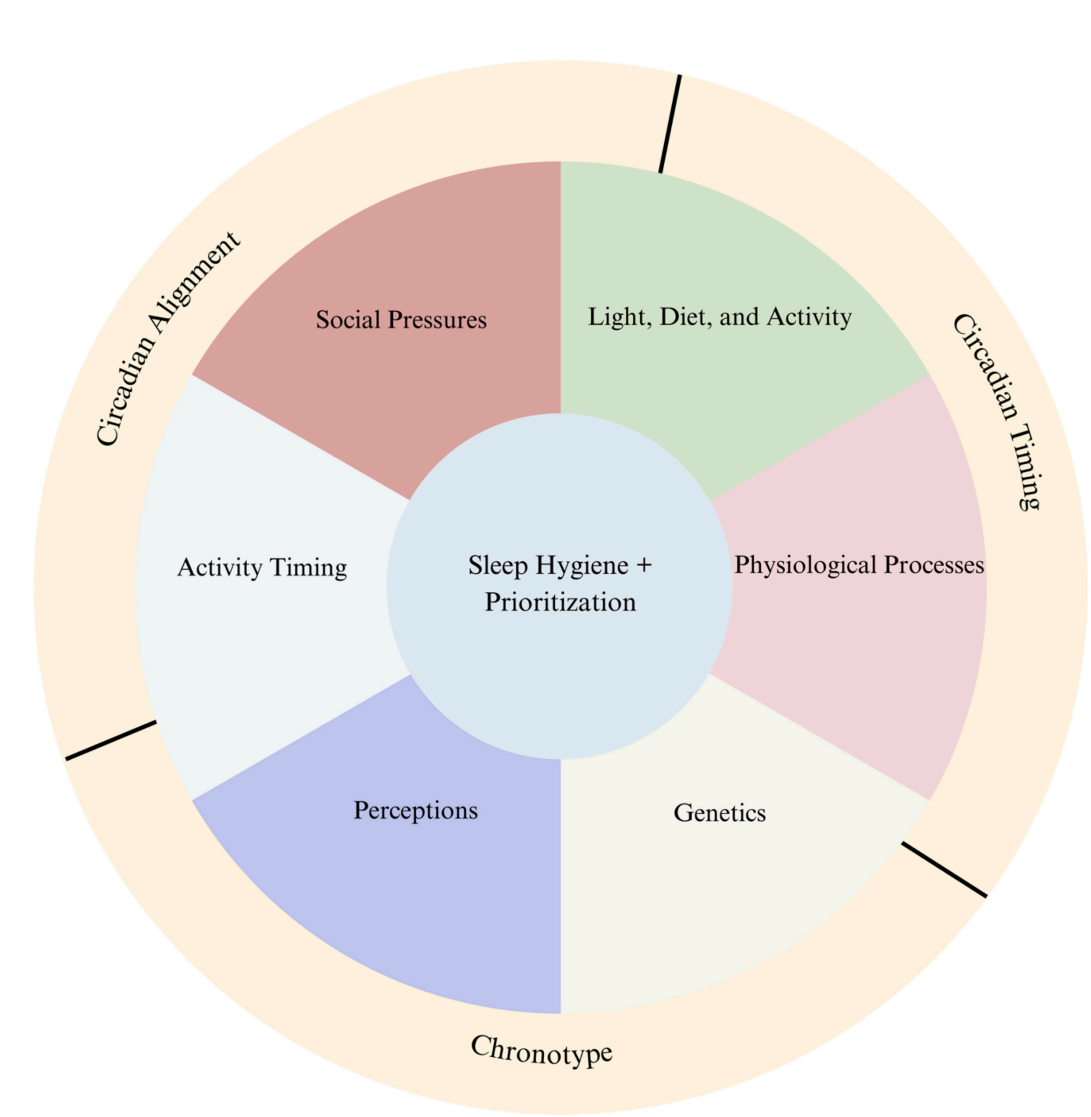
Multidimensional influences on circadian sleep health dimensions.

Given its complexity, multiple measurement approaches assessing various domains of circadian sleep health are needed to gain a more complete understanding of an adolescent's circadian sleep health profile. For example, a researcher trying to understand circadian sleep health might (a) have the adolescent complete self-report measures of their perceived chronotype; (b) measure night-to-night variability in sleep and circadian timing patterns through actigraphy; and (c) derive their biological circadian sleep timing via measurement of DLMO. Clinicians and researchers who broaden their methodological approach in this manner are better able to see the “full picture” of their patient's circadian sleep health (see Figure 3) and reduce mono-methodological bias. Furthermore, relying on multiple modalities of measurement of circadian sleep health also affords researchers the ability to understand more nuanced relationships between circadian sleep health and outcomes of interest.

### 6.1 Treatment evidence for improving circadian sleep health

A more complete picture of circadian sleep health derived from multimodal measurement can increase the focus and applicability of interventions (see Figure 3). Previous studies have found that circadian sleep health is responsive to therapeutic manipulation, leading to improvements not only in sleep, but also across other domains of wellbeing. For example, clinical trials of TSC (Harvey and Buysse, 2017) in adult community mental health settings have shown that targeting circadian sleep health leads to greater reductions in both sleep disturbance and general psychiatric impairment compared to treatment as usual (Harvey et al., 2021). Beyond being broadly beneficial, these trials suggest that circadian timing is alterable, rather than fixed. For example, administration of TSC in 10–18-year-olds has led to reductions in weeknight-weekday sleep timing discrepancies (Gasperetti et al., 2022) as well as earlier DLMO times (Harvey et al., 2018), suggesting shifts in internal rhythms as a result of outward behaviors. A study of adolescents with short and late sleep similarly found that personalized sleep routine plans which gradually advanced bedtime, paired with bright light therapy in mornings, resulted in earlier DLMO time (0.6 ± 0.8 h) vs. control (−0.1 ± 0.8 h), yielding 1.5 ± 0.7 h earlier sleep onset and 1.2 ± 0.7 increases in sleep duration after just 2 weeks (Crowley et al., 2023). *In vivo*, camping for 2 weeks, and thus being exposed to consistent bright light in mornings and dim light in evenings, has been associated with 2-h changes in DLMO time in a small sample of younger adults (Wright et al., 2013), and outdoor commutes to school have been associated with improved next-night sleep duration, leading to calls for improved bright-light exposure during the school day in order to support circadian entrainment (Kim and Casement, 2024). These findings suggest that circadian sleep health is malleable and can improve using treatment methods, though additional research is needed to determine which interventions are most effective across developmental stages and clinical contexts.

## 7 Conclusions

Sleep is a complex human system, essential for a variety of abilities, including executive function, mood regulation, and overall health. Circadian processes, which govern daily rhythms of energy, activity, and hormonal function, influence these critical areas directly. Adolescent populations face unique biopsychosocial stressors, as outlined in the Perfect Storm Model (Carskadon, 2011; Crowley et al., 2018), which increases their risk for poor sleep health and circadian misalignment. Given this heightened vulnerability, a comprehensive understanding of adolescents' overall sleep and circadian-specific health is valuable to the treatment of specific impairments (behavioral, biological, medical, etc.). Tailoring treatment to the full picture of each individual necessitates an assessment methodology which incorporates multiple dimensions of circadian sleep health, while also drawing on multiple methodologies of measurement, including self-report questionnaires, behavioral data from actigraphy, and biological markers such as dim light melatonin onset (DLMO).

Continued research examining adolescent circadian sleep health would greatly benefit from a multimodal approach that explores the interaction of different circadian dimensions on circadian sleep health. Current studies utilizing a multimodal approach have been able to examine interactions between mental health, social jetlag, and circadian sleep health among healthy adolescents to suggest multiple potential treatment targets (Duraccio et al., 2023). Similar study designs have included clinical populations as well, examining the relationship between mental wellbeing and circadian sleep health across various sleep and circadian domains (Kuula et al., 2022). These multimodal successes suggest that a more complete understanding of various variables related to circadian sleep health can better inform interventions aimed at improving adolescent health.

Although not fully comprehensive due to rapid innovation and continued application of methods to younger age groups, this paper represents the first effort to consolidate and catalog the basic measures of circadian sleep health across a variety of modalities, serving as a centralized resource for researchers and practitioners. We anticipate resources will continue to expand upon this foundation as more cost-effective and stronger methodological approaches to measuring circadian factors are developed.
